# Spatial distribution of incomplete immunization among under-five children in Ethiopia: evidence from 2005, 2011, and 2016 Ethiopian Demographic and health survey data

**DOI:** 10.1186/s12889-020-09461-3

**Published:** 2020-09-05

**Authors:** Mequannent Sharew Melaku, Araya Mesfin Nigatu, Wondewossen Zemene Mewosha

**Affiliations:** 1 Department of Health Informatics, Institute of Public Health, Wollo University, Dessie, Ethiopia; 2 Department of Health Informatics, Institute of Public Health, University of Gondar, Gondar, Ethiopia

**Keywords:** Spatial distribution, Incomplete immunization, Associated factors, Ethiopia

## Abstract

**Background:**

An estimate of 2–3 million children under 5 die in the world annually due to vaccine-preventable disease. In Ethiopia, incomplete immunization accounts for nearly 16% of under-five mortality, and there is spatial variation for vaccination of children in Ethiopia. Spatial variation of vaccination can create hotspot of under vaccination and delay control and elimination of vaccine preventable disease. Thus, this study aims to assess the spatial distribution of incomplete immunization among children in Ethiopia from the three consecutive Ethiopia demographic and health survey data.

**Method:**

A cross-sectional study was employed from Ethiopia demographic and health survey (2005, 2011and 2016) data. In total, 7901mothers who have children aged (12–35) months were included in this study. ArcGIS 10.5 Software was used for global and local statistics analysis and mapping. In addition, a Bernoulli model was used to analyze the purely spatial cluster detection of incomplete immunization. GWR version 4 Software was used to model spatial relationships.

**Result:**

The proportion of incomplete immunization was 74.6% in 2005, 71.4% in 2011, and 55.1% in 2016**.** The spatial distribution of incomplete immunization was clustered in all the study periods (2005, 2011, and 2016) with global Moran’s I of 0.3629, 1.0700, and 0.8796 respectively. Getis-Ord analysis pointed out high-risk regions for incomplete immunization: In 2005, hot spot (high risk) regions were detected in Kefa, Gamogofa, KembataTemibaro, and Hadya zones of SNNPR region, Jimma zone of Oromiya region. Similarly, Kefa, Gamogofa, Kembatatemibaro, Dawuro, and Hadya zones of SNNPR region; Jimma and West Arsi zones of Oromiya region were hot spot regions. In 2016, Afder, Gode, Korahe, Warder Zones of Somali region were hot spot regions. Geographically weighted regression identified different significant variables; being not educated and poor wealth index were the two common for incomplete immunization in different parts of the country in all the three surveys.

**Conclusion:**

Incomplete immunization was reduced overtime across the study periods. The spatial distribution of incomplete immunization was clustered and High-risk areas were identified in all the study periods. Predictors of incomplete immunization were identified in the three consecutive surveys.

## Background

Vaccination has proved to be one of the most cost-effective health interventions worldwide, through which many childhood diseases have been prevented [1]. Childhood immunization is considered to be one of the most important health indicators of a healthy childhood since, it assures protection from major childhood diseases consequently, it prevents millions of deaths and cases of disability worldwide [2].

The World Health Organization began the Expanded Program on Immunization with the purpose of regulating eight vaccine preventable childhood diseases like: tuberculosis, diphtheria, pertussis (whooping cough), tetanus, polio, measles, hepatitis B and hemophiles influenza type B (Hib). In line with the WHO’s references, Currently, the national EPI in Ethiopia aims to immunize children between the ages of 0 and 23 months against eight vaccine-preventable childhood diseases: pentavalent, BCG, polio, and measles [3].

In 1980, 84% of the world’s children lived in countries where immunization coverage was less than 50%, in contrast in 2006, 57% of children lived in countries with greater than 80% DTP3 coverage [4]. Approximately 29% of deaths in children under five are vaccine preventable [5]. Immunization service is designed as a key strategy to reduce under five mortality in Millennium development goal and sustainable development goal [6, 7]. The second theme of sustainable development goal three is aiming to end preventable deaths of newborns to at least minimize 12 per 1000 live births and under 5 children to. at least minimize 25 per 1000 live births could be achieved through consistent implementation of immunization service in all countries by designing different strategies based on the setup of each countries [8]. Efforts in attaining this goal focus on developing countries, which account for over three fourth of child deaths [9].

Despite the efforts to improve vaccination services, around 27 million infants and 40 million women were not vaccinated against measles or tetanus in 2007, consequently, 2–3 million children are dying annually from easily vaccine-preventable diseases, and many more fall ill [1, 10]. An estimated 23.2 million children were leftovers unvaccinated globally, of which 15.3 million (65%) reside in eight countries mainly in Africa [11]. In 2012 WHO revealed that around 1.5 million children under five years of age worldwide died from the vaccine-preventable disease [1, 2].

In 2017, around 19.9 million infants in the world did not get routine immunization services (pentavalent, measles and polio); about 60% of these children from the above figure have been living in 10 countries (Angola, Ethiopia, India, Indonesia, Iraq, Nigeria, Afghanistan, Democratic Republic of Congo, Pakistan and South Africa) [12].

Ethiopia is a country, in sub-Saharan Africa and has extremely low immunization coverage in which nearly 1 million children were estimated to be unvaccinated and about 16% under-five mortality has been attributed to vaccine-preventable diseases in the country; although vaccination coverage has been improved in Ethiopia, still millions of children remain unvaccinated and thousands of deaths occur every year [11]. Even though achievements observed in the reduction of under-five mortality rates, about 190,000 children are still dying each year [13].

According to the Ethiopia Demographic and Health Survey (EDHS) reports, vaccination coverage showed slight increments for the consecutive EDHS periods,14% in 2000, 20% in 2005, 24.3% in 2011, 38.5% in 2016 [10, 14, 15]. Even though the immunization coverage of the country increased from wave to wave of EDHS still it is less than half of the national target. In contrast to the existing situation in the country reported by WHO, UNICEF and EDHS the HMIS report of EFY 2006 showed that the coverage of Pentavalent 3, PCV3 and Measles vaccine coverage has reached 91.1, 85.7, and 86.5% respectively. In addition, fully immunized children under one year of age also reached 82.9% in EFY 2006 [13].

Different factors are frequently associated with incomplete immunization status both in developed and developing regions [16–27]. Limited resources, limited infrastructure coverage, and illiteracy contribute to Sub-Saharan countries to have high incomplete immunization [13].

There is a longstanding understanding about disease occurrence is not a random phenomenon, but rather it is a result of specific set of interactions. Spatial epidemiology is relatively young field of science but has its origin with John Snow in the mapping of the cholera outbreak in broad street, London in 1854 [28]. Spatial analysis in Epidemiology roots its foundation in the basic principles of descriptive Epidemiology, that it tries to identify the geographical or place distribution of disease occurrence, and whether the disease distribution is place dependent or whether there is geographical dependence in disease occurrence between neighboring regions or any pair of regions. The base of any spatial studies is the Tobler‟s first law of geography by Waldo Tobler (1930), which states “Everything is related to everything else, but near things are more related than distant things “ [29].

Previous spatial studies have revealed the existence of spatial variation for vaccination [30–32]. Studies of specific vaccines in Ethiopia have also revealed the presence of spatial variation for vaccination [33–35]. However spatial studies on basic vaccination completion status of children is limited and Spatial variation of incomplete immunization can delay control and elimination of vaccine preventable disease even in countries with high average nationwide vaccination coverage [36, 37]. Understanding the area-based heterogeneity of incomplete immunization is a footstep for evidence-based decision-making in vaccine preventable disease prevention and control program and detecting spatial variation is useful to recognize gaps in the performance of immunization programme that could not be identified through the routine monitoring of vaccination coverage [37]. However, studies are limited on the spatial pattern of incomplete immunization for childhood in Ethiopia. Hence, this study aimed to explore the spatial pattern of incomplete immunization among children.

## Methods

### Study design and settings

A nationally representative population-based cross-sectional survey (2005, 2011, and 2016) study was used to show the spatial distribution of incomplete immunization in Ethiopia.

**Fig. 1:**
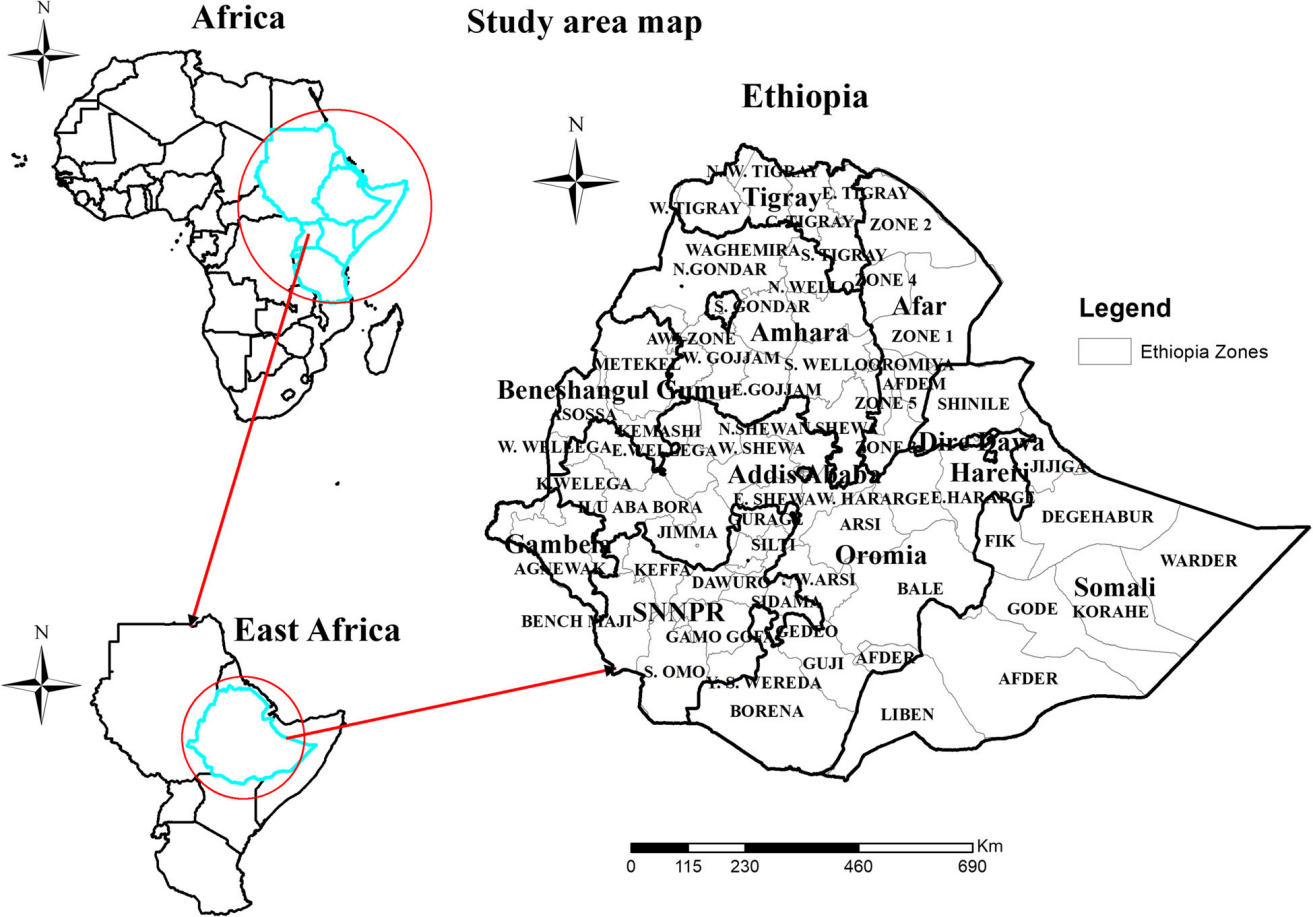
Map of the study area [Shape file source: CSA, 2013; URL: https://africaopendata.org/dataset/ethiopia-shapefiles]

Ethiopia is found in the horn of Africa covering 1,104,300 km^2^ and ranks tenth in Africa in land coverage [38]. Ethiopia is a country with a great geographical diversity ranging from peaks up to 4550 m above sea level down to a depression of 110 m below sea level [13]. Ethiopia has nine administrative regions and two City administrations (Fig. 1) divided into 68 zones, 817 districts, 16,253 kebeles (the smallest administrative unit of the country) at the end of 2010 EFY.

Projections from the 2007 population and housing census estimate a total population of 108,805,142 in 2018. According to the 2007 census a majority of the population (83.6%) was living in rural areas, the average household size was 4.7 persons, the population age was predominately young: 44% were under 15 years, over half, 52% were between 15 to 65 years [13]. Ethiopia has been achieved a remarkable achievement in health facility coverage, which revealed that 17,685 health posts, 392 hospitals, 3962 health centers were available at the end of EFY 2010 in all health facilities immunizations service is provided at least 2 days a week [39, 40].

The EDHS waves were conducted from April 27 to August 30, 2005, December 27, 2010, to June 3, 2011, and January 18, 2016, to June 27, 2016 [10, 14, 15]. All women aged 15 to 49 years who had under-five children and women aged 15 to 49 years who had children started immunization were considered as the source population and study population respectively. In addition**,** Mothers who had children aged 12 to 35 months and whose children had been started vaccination were included and Mothers who had children aged 12 to 35 months and whose children had incorrectly registered age, incomplete data elements were excluded.

### Sample size estimation, sampling methods and procedure

A total of 7901 children aged 12 to 35 months samples were drawn from three successive EDHSs periods (1373 in 2005, 3333 in 2011, and 3195 in 2016) considered for the entire analysis.

**Fig. 2:**
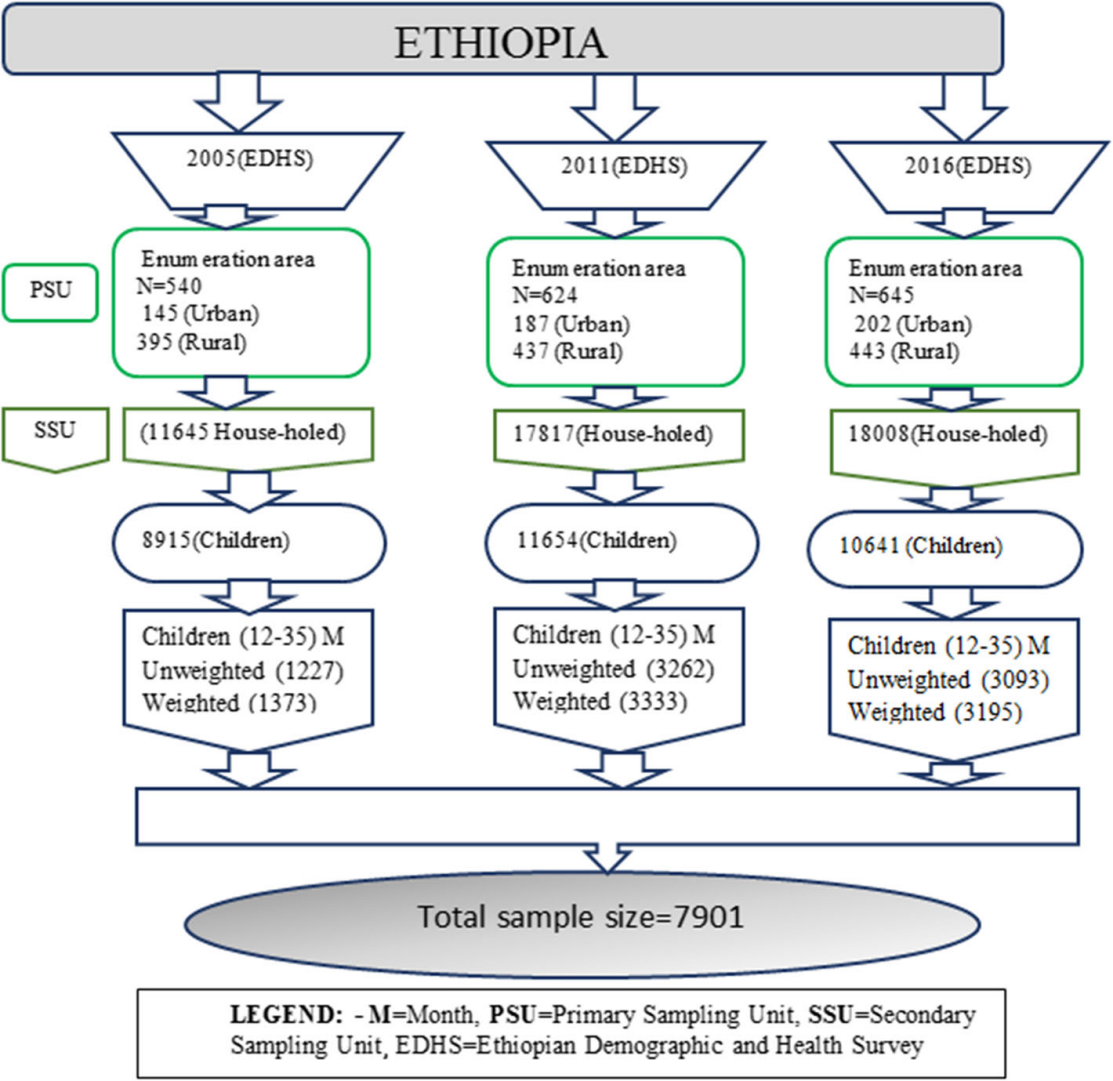
Sampling procedure

A two-stage probability sampling method which was stratified by geographic region and by urban/rural areas within each region that entirely covers the target population of Ethiopia was used [10, 14, 15]. The overall probability of selection of a household was different from cluster to cluster (Fig. 2).

### Data source and methods

The questionnaire included socio-demographic, socioeconomic, and vaccination information. A stratified two-stage cluster sampling procedure was employed for all three surveys. Location data (latitude and longitude coordinates) were also taken from the selected enumeration area. The survey datasets and location data were accessed through the web page of the International DHS Program after subscription and being authorized by International Classification of Functioning, Disability, and Health (ICF).

### Data extraction

After accessing the data from MEASURE DHS website data extraction, data weighting, data cleaning and recoding were carried out using STATA version 14.1 (Stata Corp. Statistical Software).

### Operational definition

- Incomplete immunization (partially immunized): is defined as children who started vaccination and missed at least one dose of vaccination from (one dose of BCG, three doses of polio from four doses, three doses of pentavalent, and one dose of measles) at any time instance between 0 and 23 months.
- Complete immunization: when children had vaccinated for BCG, three doses of pentavalent, at least three dose of polio, and one dose of measles.

### Study variables

#### Dependent variable

Incomplete immunization a binary outcome which was categorized as 1 = yes (children who had not completed the full dose of vaccination) and 0 = no (for who had taken the full dose of vaccination).

#### Independent variables

Socio-demographic factors; mothers age: categorized into 15-24 years, 25-34 years and 35–49; mothers educational level; categorized into not educated, primary, secondary and above; marital status: categorized into married and not married; sex of household head; categorized into male and female; wealth index: categorized into poor, middle and rich; occupation: categorized into not working, professional work and have no professional work; religion: orthodox, Muslim, Catholic, Protestant and others; relationship with household head: head, wife, daughter and others; number of living children: categorized into 1–3, 4–6 and 7 and above.

### Data analysis

Descriptive and summary statistics such as frequency table and cross tabulation were handled using STATA version 14.

### Spatial autocorrelation analysis

The spatial autocorrelation (Global Moran’s I) statistic was held in order to assess the pattern of incomplete immunization whether it was dispersed, clustered or randomly distributed in the study area. When computed, Moran’s I values near to − 1 showed that the event was dispersed, whereas when Moran’s I near to + 1 showed that the event was clustered and event distributed randomly if Moran’s I value zero. A statistically significant Moran’s I (*p* < 0.05) lead to confirm the existence of spatial autocorrelation [41].

### Local Moran’s I analysis

Local Moran’s I analysis detects outliers of the cluster which is impossible to detect using Getis-Ord analysis and hot spot as well as cold spot zones in order to make sure that the consistency of findings by Getis-Ord analysis. Local Moran’s I measure positively correlated high-high (hotpot) and low-low (cold spot) clusters. If a higher value is surrounded primarily by lower values, and a lower value is surrounded primarily by higher values it is said to be an outlier. A positive value for ‘I’ indicated that a case had neighboring cases with similar values, such type of case was part of a cluster. A negative value for ‘I’ indicated that a case is surrounded by cases with dissimilar values; this case is an outlier [42, 43].

### Getis-Ord Gi* hot spot and cold spot analysis

Getis-Ord Gi* statistics had been calculated to measure how spatial autocorrelation differs through the study location by computed Gi* statistics for each area. Z-score was calculated to ensure the statistical significance of clustering at *p*-value < 0.05 at 95 CI. If z-score is between − 1.96 and + 1.96, the p-value must be greater than 0.05 and vice versa. If p-value is less than − 1.96, it is declared as cold spot and if greater than + 1.96 it is declared as hotspot areas. Statistical output with high Gi* indicates “hot spot” areas, whereas low Gi* means a “cold spot’ areas [44].

### Interpolation

The spatial interpolation technique was applied to predict the unsampled data using the data feed by sampled data. Ordinary kriging interpolation method was used to predict the risk of incomplete immunization in Ethiopia in all the study periods [45].

### Spatial scan statistical analysis

SaTScan analysis further intensifies the finding which detected by Getis-Ord analysis which is strong to detect hot spot regions and enables to reports confirmatory findings for the analysis. The spatial Scan statistics method is used to spot local clusters and has higher power than other available spatial statistical methods [46, 47]. Spatial Scan statistical analysis was handled to test the occurrence of statistically significant high rate clusters of incomplete immunization among children using a Bernoulli model by Kuldorff’s SaTScan version 9.6 software. Children who had been incompletely immunized were considered as cases and children who were took the full dose of immunization as controls. A spatial scan statistic used a scanning window (the population at risk) in the shape of a circle, which moves across the study region [46]. For the purposes of this study, the size of the scan window was sated at 25% of the study population, to scan for small clusters which may possibly be more amenable to interventions. The likelihood function is maximized over all windows, and the window with the maximum likelihood ratio constitutes the most likely cluster [47].

When generating secondary clusters, selection of non-overlapping options in SaTScan version 9.6 was employed. Clusters with large likelihood ratios were identified and the significance of the recognized clusters depends on the likelihood ratio test whose *p*-value is generated by applying Monte Carlo replications. The number of Monte Carlo replications was slated to 999 to ensure adequate power for defining clusters and a p-value less than 0.05 was considered statistically significant; the specific locations of clusters were assessed using relative risks (RRs) [47]. ArcGIS software version 10.4 was used to map the cluster and attribute of incomplete immunization produced by SaTScan™.

### Spatial regression

Spatial regression has both local and global analysis techniques as usual of GIS models [48–50]. Therefore, first we had handled global geographical regression models and then local geographical analysis in order to ensure that the variability of coefficients across each enumeration area of the respective study period [51–53]. We have checked assumptions of spatial regression using expletory regression with the respective tests. The normality assumption was checked for residuals using Jarque-Bera test. As residuals are not spatially auto correlated, confirming koenker Bp test was done to check the model if under gone for geographically weighted regression or not. Geographically weighted regression was executed using GWR version 4 software. We had also checked the six checks which recommended for a model undergone for spatial regression [54, 55] this are: coefficients have the expected sign, no redundancy among model explanatory variables, coefficients are statically significant, strong adjusted R^2^ values and the above two conditions stated before. Variables with *p*-value less than 0.05 were selected and described based on their coefficients.

### Ethical consideration

The survey process had been gone through the required ethical clearance procedures [10, 14, 15]; however, permission to use the dataset has been granted by the Measure DHS program through legal registration. EDHS (2005, 2011, and 2016) data was used which is available on the public domain through the Measure DHS website (www.measuredhs.com) [10, 14, 15]. Accordingly, the investigators had requested permission to use the data set on the measure DHS website about the spatial distribution of incomplete immunization among children in Ethiopia on January 13, 2019. The study was approved by the Institutional Ethical Review Committee Board of the University of Gondar and ethical clearance was obtained from the board. Due to the retrospective nature of the study, informed consent had been waived.

## Results

### Socio demographic characteristics of the participant

Among mothers who were participating in this study 1270(92.5%) were married, 1029 (74.98%) were not educated. In 2005 EDHS, in 2011 3102 (93%) were married, 2222 (66.68%) were not educated. In 2016 3000 (93.9%) were married, 1960 (61.33%) were not educated. The mean age of children was 22.87 ± 7.12 in 2005, 23.63 ± 7.09 in 2011 and 22.79 ± 7.07 months in 2016 with a minimum value of 12 and max 35 months in all the study periods. The mean age of mothers was 29.28 years±7.07 in 2005, 28.96 years±6.52 in 2011, 29.25 years±6.49 in 2016 (Table 1).

**Table 1:** Socio-demographic characteristics of the participants, 2005, 2011, 2016 EDHS

Variables	2005 EDHS *n* = 1373 Frequency (%)	2011 EDHS *n* = 3333 Frequency (%)	2016 EDHS *n* = 3195 Frequency (%)
Mothers education			
No education	1029 (74.98)	2222 (66.68)	1960 (61.33)
Primary	263 (19.15)	955 (28.64)	955 (29.89)
Secondary higher	81 (5.87)	156 (4.68)	280 (8.78)
Religion			
Orthodox	648 (47.2)	1378 (41.37)	1200 (37.56)
Catholic	13 (0.91)	29 (0.86)	44 (1.39)
Protestant	271 (19.75)	740 (22.23)	735 (23.01)
Muslim	417 (30.38)	1115 (33.48)	1152 (36.04)
Others	24 (1.75)	71 (2.06)	64 (1.99)
Wealth index			
Poor	578 (42.1)	1419 (42.58)	1399 (43.78)
Middle	279 (20.3)	699 (20.97)	699 (21.88)
Rich	516 (37.6)	1215 (36.45)	1097 (34.34)
Marital status			
Married	1270 (92.5)	3102 (93)	3000 (93.9)
Not married	103 (7.5)	231 (7)	195 (6.1)
Age			
15–24	362 (26.35)	844 (25.34)	742 (23.21)
25–34	655 (47.76)	1729 (51.86)	1722 (53.92)
35–49	356 (25.89)	760 (22.8)	731 (22.87)
Relationship with household head			
Head	117 (8.49)	370 (11.1)	341 (10.66)
Wife	1154 (84)	2713 (81.6)	2562 (80.19)
Daughter	50 (3.7)	143 (4.1)	161 (5.05)
Others	52 (3.8)	107 (3.2)	131 (4.1)
HH head Sex			
Male	1214 (88.45)	2860 (85.82)	2737 (85.66)
Female	159 (11.55)	473 (14.18)	458 (14.34)
Mother Occupation			
Not working	885 (64.6)	1472 (44.16)	1661 (51.98)
Professional work	447 (32.6)	1569 (47.06)	1211 (37.92)
Not profess work	39 (2.9)	292 (8.78)	323 (10.1)
No of living children			
1–3	714 (52)	1842 (55.28)	1694 (53.01)
4–6	474 (34.5)	1110 (33.3)	1102 (34.49)
7+	185 (13.5)	381 (11.42)	399 (12.51)

HH = households

**Fig. 3:**
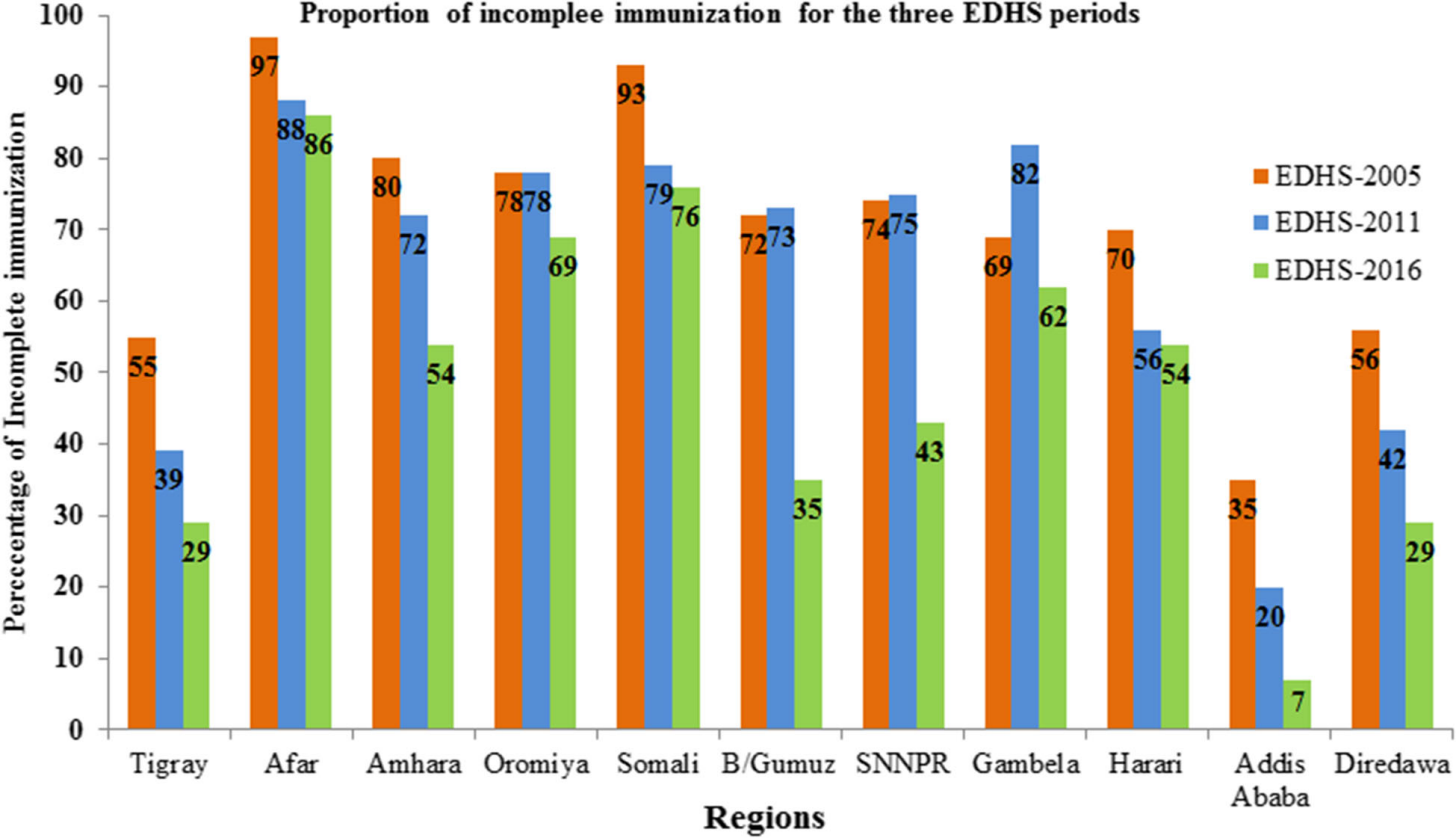
Regional variation of incomplete immunization in Ethiopia

In this study the proportion of children incompletely immunized was 74.6% in 2005, 71.4% in 2011, and 55.1% in 2016. Incomplete immunization varied from 33% in Addis Ababa, 93% in Somali and 97% in Afar by 2005. For the period 2011, incomplete immunization varied from 20% in Addis Ababa and 86% in Afar. Whereas in 2016, 7% in Addis Ababa and 83% in Afar. In the three consecutive waves of EDHS survey Benshangul-Gumz and SNNPR had shown a great reduction of incomplete immunization which was 37, 31% respectively (Fig. 3).

### Elements of complete immunization

Components of complete immunization are BCG, polio 1, polio 2, polio 3, pentavalet 1, pentavalet 2, pentavalet 3, and measles vaccinations. Accordingly, the description of each component in each survey years: - vaccinated for BCG was 76.55% in 2005, 77% in 2011, 83% in 2016 (Table 2).

**Table 2:** Components of complete immunization in Ethiopia, 2005, 2011, 2016 EDHS

Vaccine Type	2005 n = 1373		2011 n = 3333		2016 n = 3195	
	Vaccinated		Vaccinated		Vaccinated	
	Yes	No	Yes	No	Yes	No
BCG	1051 (76.55)	322 (23.45)	2566 (77)	767 (23)	2652 (83)	543 (17)
Penta 1	968 (70.5)	405 (29.5)	2375 (71.25)	958 (28.75)	2726 (85.4)	469 (14.6)
Polio 1	1352 (98.46)	21 (1.54)	3219 (96.58)	114 (3.42)	3066 (96)	129 (4)
Penta 2	792 (57.67)	581 (42.33)	1975 (59.24)	1358 (40.76)	2366 (74.1)	829 (25.9)
Polio 2	1204 (87.68)	169 (12.32)	2830 (84.9)	503 (15.1)	2739 (85.73)	456 (14.27)
Penta 3	559 (40.69)	814 (59.31)	1404 (42.14)	1929 (57.86)	1918 (60.03)	1277 (39.97)
Polio 3	891 (64.9)	482 (35.1)	1977 (59.3)	1356 (40.7)	2107 (65.94)	1088 (34.06)
Measles	632 (46.05)	741 (53.95)	2194 (65.83)	1139 (34.17)	2116 (66.23)	1079 (33.77)

### Spatial analysis

#### Spatial autocorrelation analysis

**Fig. 4:**
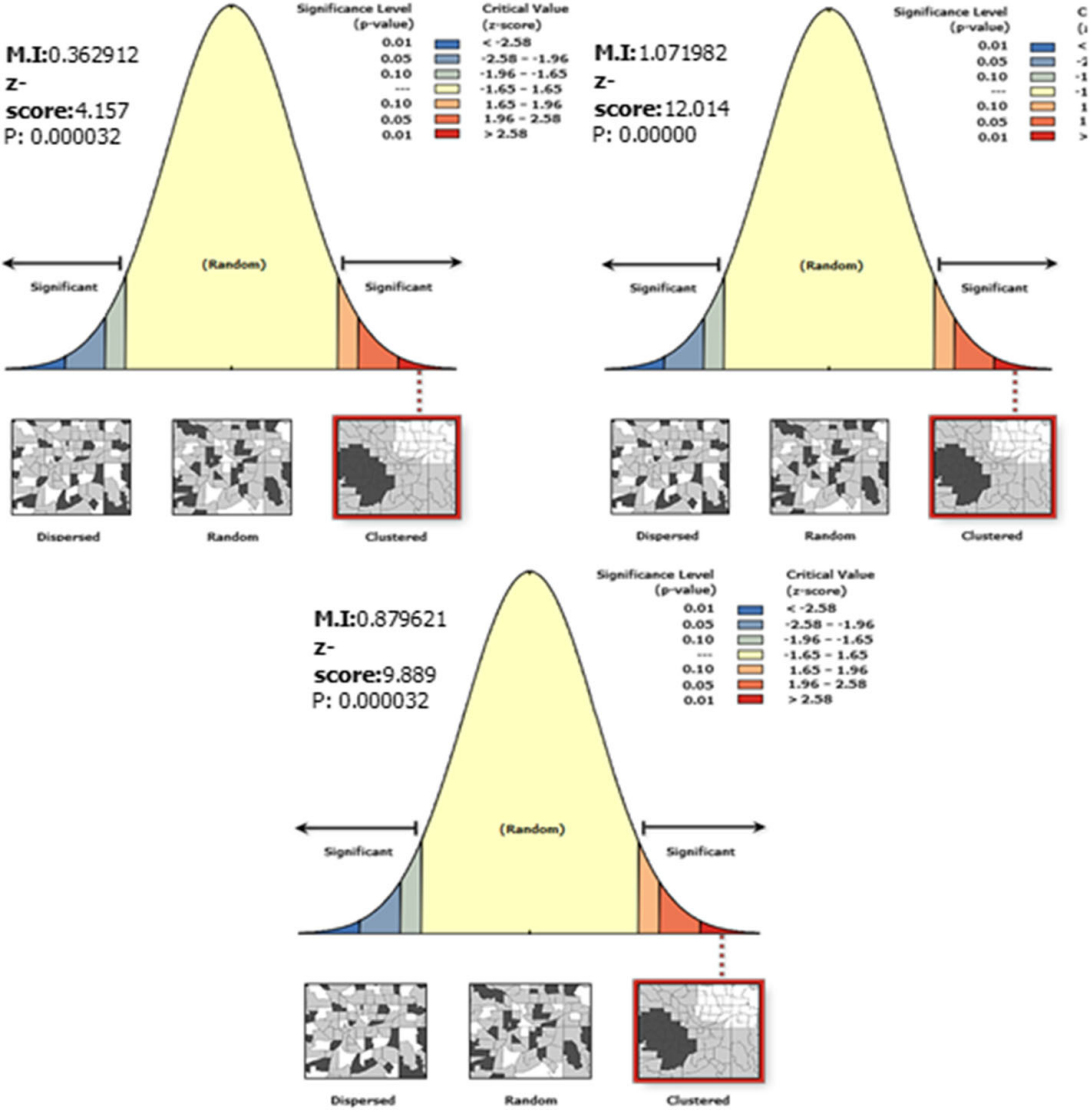
Spatial autocorrelation of incomplete immunization in Ethiopia, EDHS 2005, 2011 and 2016. The right panel represents for 2005 EDHS, the middle panel represents for 2011 EDHS, and the left panel represents for 2016 EDHS. The right part of the panel indicates dispersed, the central part of the panel indicates random, and the left part of the panel indicates clustered. In this study incomplete immunization is clustered in all periods. The clustered pattern on the right side of the panels indicates that high rate of incomplete immunization over the study periods the output has automatically generated keys on the upper right side of the panel as well as in the underneath reveals that: there is less than 1% likelihood that this clustered pattern could be the result of random chance. [Shape file source: (CSA, 2013; https://africaopendata.org/dataset/ethiopia-shapefiles); Map output: Own analysis using ArcGIS 10.7 Software]

**Fig. 5:**
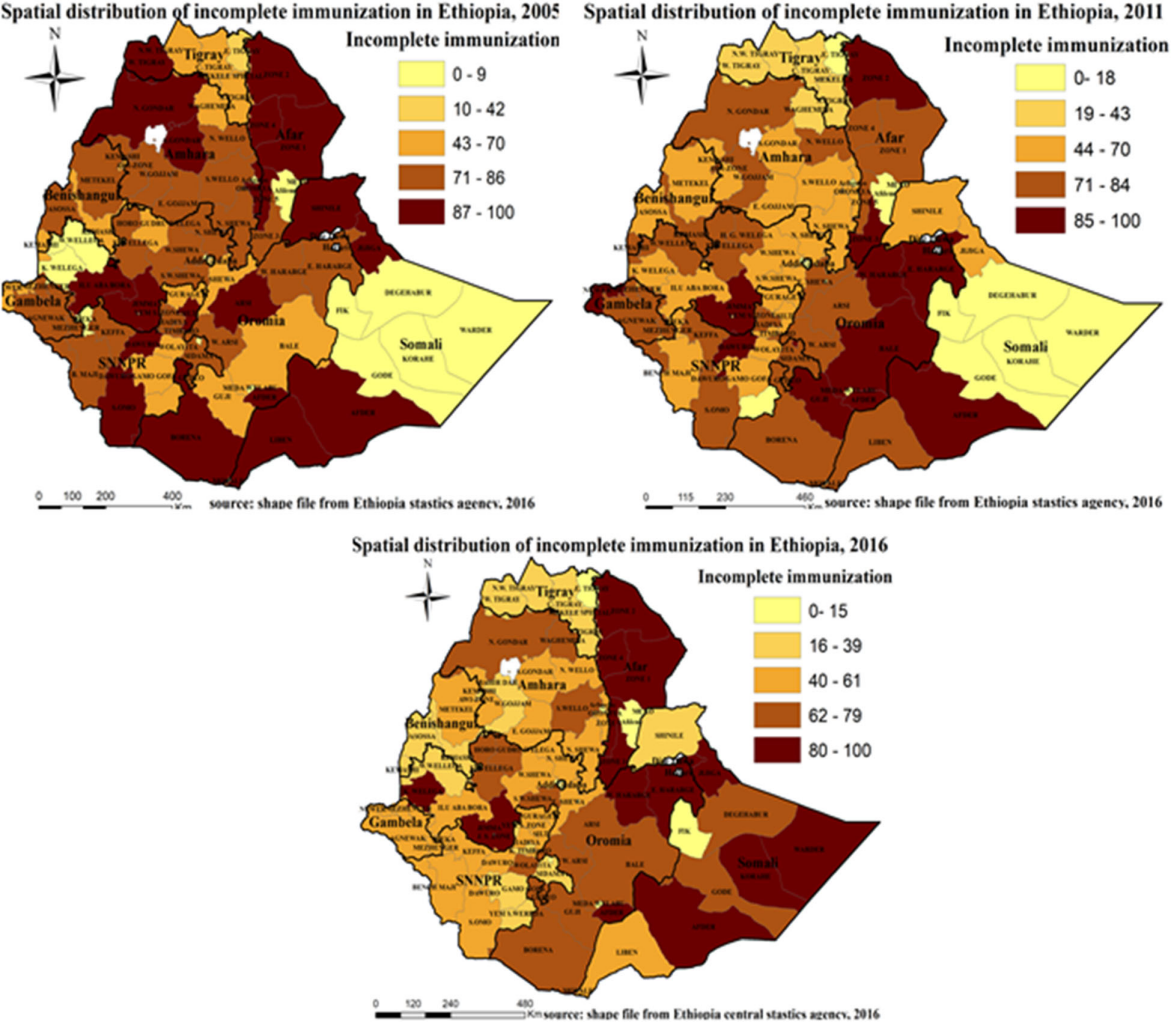
Spatial distribution of incomplete immunization in Ethiopia, EDHS 2005, 2011 and 2016. Spatial distribution of incomplete immunization in Ethiopia from Ethiopian demographic and health survey. The right panel represents for 2005 EDHS, and the left panel represents for 2011 EDHS the right bottom panel represents for 2016 EDHS. In the figure the damp gray color indicates very high clustering, the bright gray color indicates high clustering, the damp yellow color indicates moderate clustering, the bright yellow color indicates low clustering, and the whitish yellow color indicates very low clustering of incomplete immunization cases. The thick white black line indicates regional borders while the thin white black line indicates zonal borders. [Shape file source: (CSA, 2013; URL: https://africaopendata.org/dataset/ethiopia-shapefiles); Map output: Own analysis using ArcGIS 10.7 Software]

The spatial distribution of incomplete immunization was clustered in all the study periods at zonal level (Fig. 4). The spatial auto correlation global Moran s I index of each EDHS survey was (0.362912 (*p*-value < 0.001) in 2005, 1.071982 (p-value < 0.001) in 2011, 0.879621 (p-value < 0.001) in 2016) (Table 3). The spatial distribution of incomplete immunization revealed that the presence of significant variation across Ethiopia zones. The highest incomplete immunization was spatially clustered in North Gondar, South Gondar, Argoba, and Oromiya special zones of the Amhara region, Zone 1, Zone 2, Zone 4 of Afar region, Iluababora, Jimma, Arsi,zones of Oromiya region in 2005, Zone 2 and Zone 3 of Afar region, East Harerige, West Harerige, Bale, Jimma, Afder, Guji, and Medawalabu zones of Oromiya region, Afder zone of Somali region, Newuer zone of Gambella region, and Dawuro zone of SNNPR in 2011, Zone 1, Zone 2, Zone 3, and Zone 4 of Afar region, East Harerige, West Harerige, Jimma, and Kelem Wollega of Oromiya region, Afder, Korahe, Jigjiga, Warder zones of Somali region in 2016 (Fig. 5).

**Table 3:** Summary of spatial autocorrelation of incomplete immunization in Ethiopia, 2005, 2011, 2016

EDHS study Periods	Observed Moran ‘s I	Expected Moran’s I	Z-score	P-value
2005	0.362912	−0.011494	4.157	< 0.001
2011	1.07198	−0.01802	12.014	< 0.001
2016	0.87962	−0.01495	9.889	< 0.001

#### Local Morans I result of incomplete immunization

**Fig. 6:**
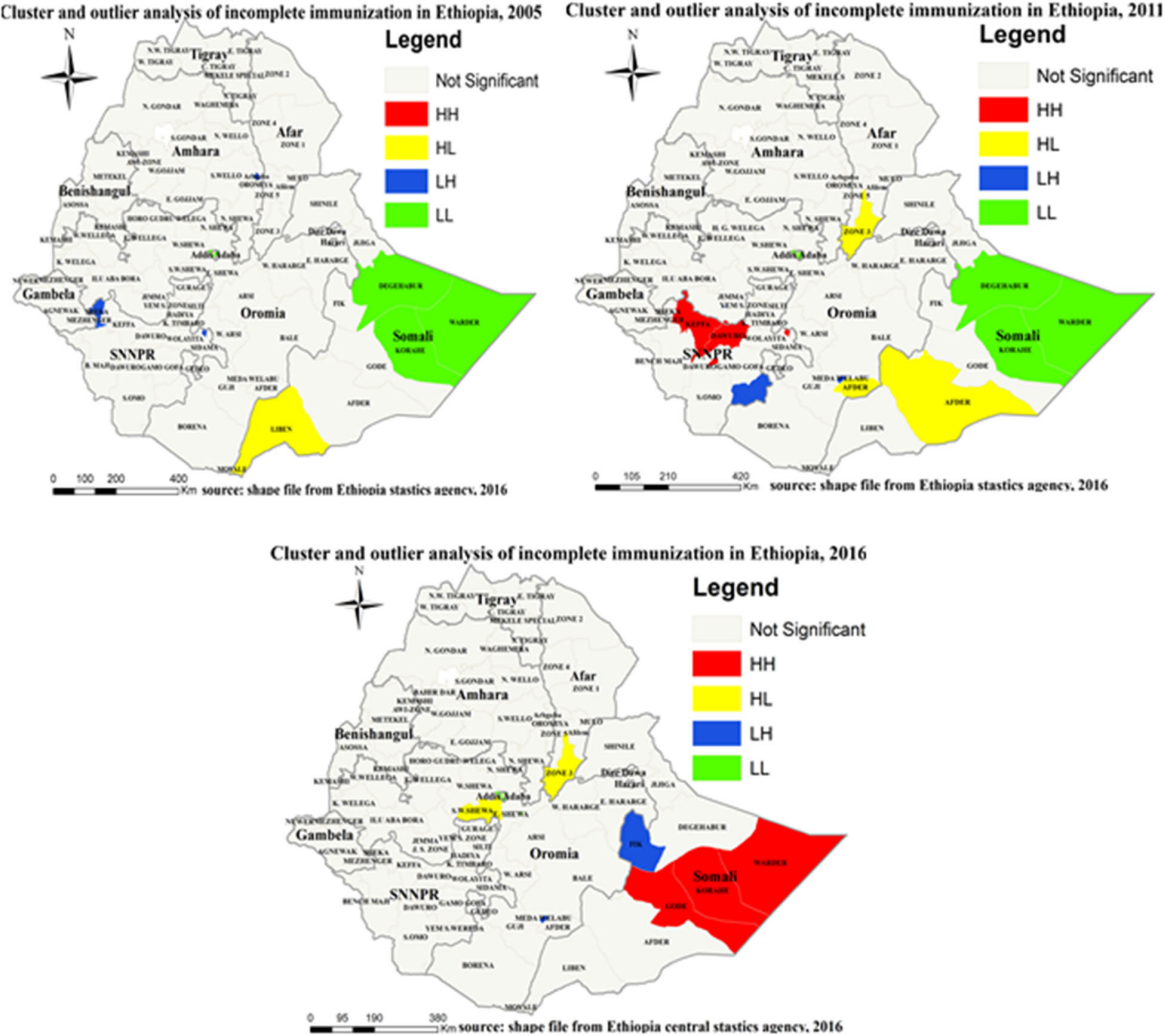
Cluster and outlier analysis of incomplete immunization in Ethiopia, EDHS 2005, 2011 and 2016. Local Moran’s I analysis result of incomplete immunization in Ethiopia from Ethiopian demographic and health survey. The right panel represents for 2005 EDHS, and the left panel represents for 2011 EDHS the right bottom panel represents for 2016 EDHS. In the figure the red color indicates high clustering, the yellow color indicates high outlier clustering, the blue color indicates low outlier clustering, and the green color indicates low clustering the fogy color indicates insignificant clustering. The thick white black line indicates regional borders while the thin white black line indicates zonal borders. [Shape file source: (CSA, 2013; URL: https://africaopendata.org/dataset/ethiopia-shapefiles); Map output: Own analysis using ArcGIS 10.7 Software]

Local Moran’s I analysis result of each survey revealed that there are significant outliers in all the surveys. High outlier (high level of incomplete immunization surrounded by low level of incomplete immunization) was observed in Liben zones of Somali region in 2005, Zone 3 of the Afar region, Afder zone of the Somali region in 2011, Zone 3 of the Afar region, South West Shewa zone of the Oromiya region, and low outlier (low level of incomplete immunization surrounded by high level of incomplete immunization) was observed in Sheka zone of the Gambella region, Arbgoba special zone of the Amhara region for 2005, Yem special zone of SNNPR, Medawalibu special enumeration area of the Oromiya region in 2011, Fik zone of the Somali region, Medawalibu special enumeration area of Oromiya the region IN 2016 (Fig. 6).

#### Gettis-OrdGi* statistics identification of incomplete immunization hot spot zones in Ethiopia

**Fig. 7:**
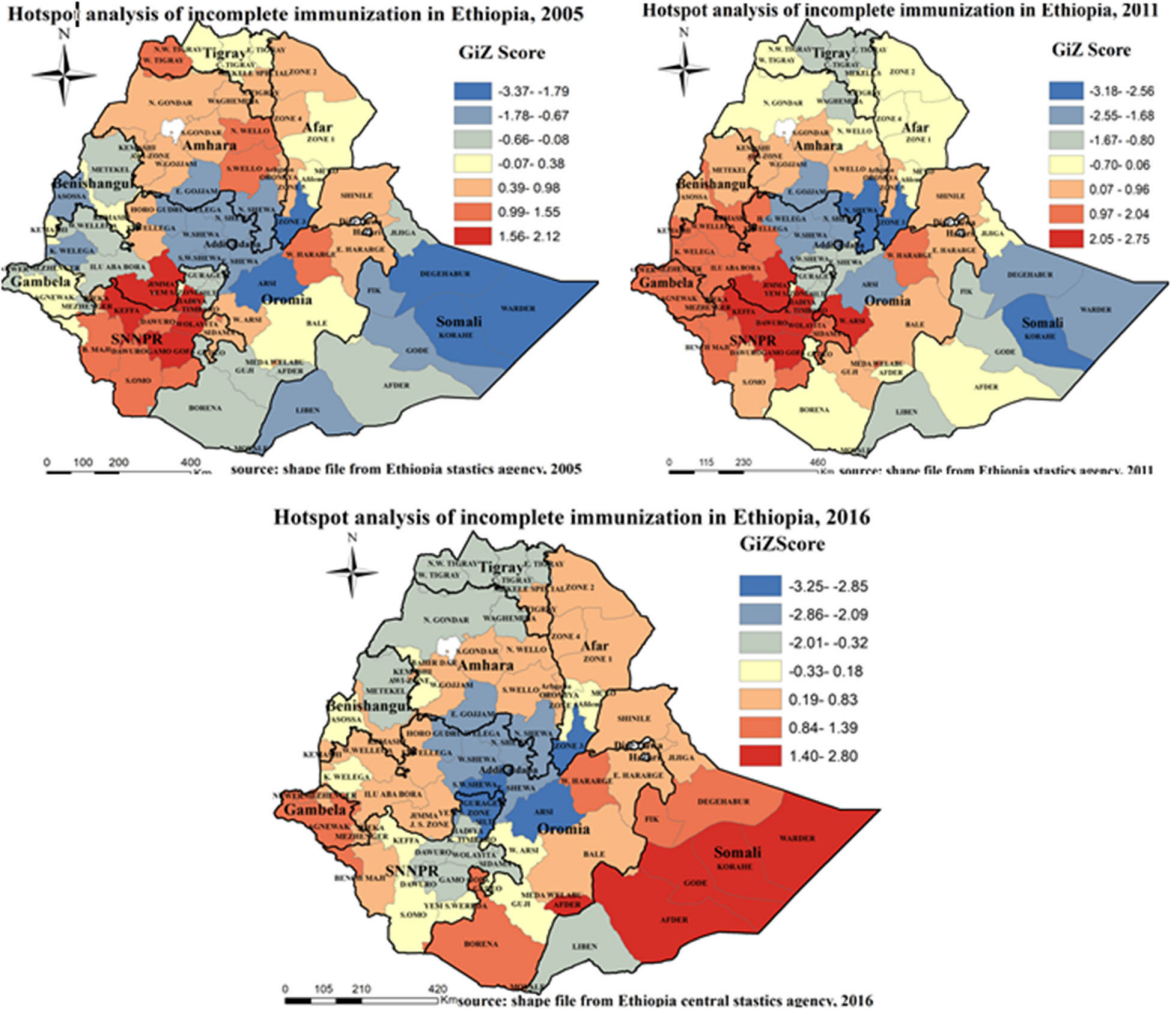
Gettis-Ord analysis of incomplete immunization in Ethiopia, EDHS 2005, 2011 and 2016. Gettis-Ord Gi* local Hotspot analysis result of incomplete immunization in Ethiopia from Ethiopian demographic and health survey. The right panel represents for 2005 EDHS, and the left panel represents for 2011 EDHS the right bottom panel represents for 2016 EDHS. In the figure the red color indicates high hotspot zones, the blue color indicates cold spot zones, the whitish yellow color indicates insignificant clustering of incomplete immunization. The thick black line indicates regional borders, while the thin white black line indicates zonal borders. [Shape file source: (CSA, 2013; URL: https://africaopendata.org/dataset/ethiopia-shapefiles); Map output: Own analysis using ArcGIS 10.7 Software]

Hotspot analysis enables to detect both extremities either high or low incomplete immunization coverage zones. Accordingly, hot spot (high risk) regions for incomplete immunization were detected in Kefa, Gamogofa, Kembata Temibaro, and Hadya zones of SNNPR region, Jimma zone of Oromiya region in 2005, Kefa, Gamogofa, Kembata temibaro, Dawuro, and Hadya zones of SNNPR region, Jimma and West Arsi zones of Oromiya region in 2011, Afder, Gode, Korahe, Warder zones of Somali region in 2016. On the other hand, Arsi zone of Oromiya region, zone 3 of Afar region, Warder, Korahe, and Degahbur zones of Somali region in 2005, North Shewa of Amhara region, zone 3 of Afar region, Korahe zone of Somali region in 2011, South West Shewa and Arsi zone of Oromiya region, zone 3 of Afar region, Gurage, Silte, Yem special zones of SNNPR region were cold spot (low risk) regions for incomplete immunization (Fig. 7).

#### Spatial interpolation of incomplete immunization in Ethiopia

**Fig. 8:**
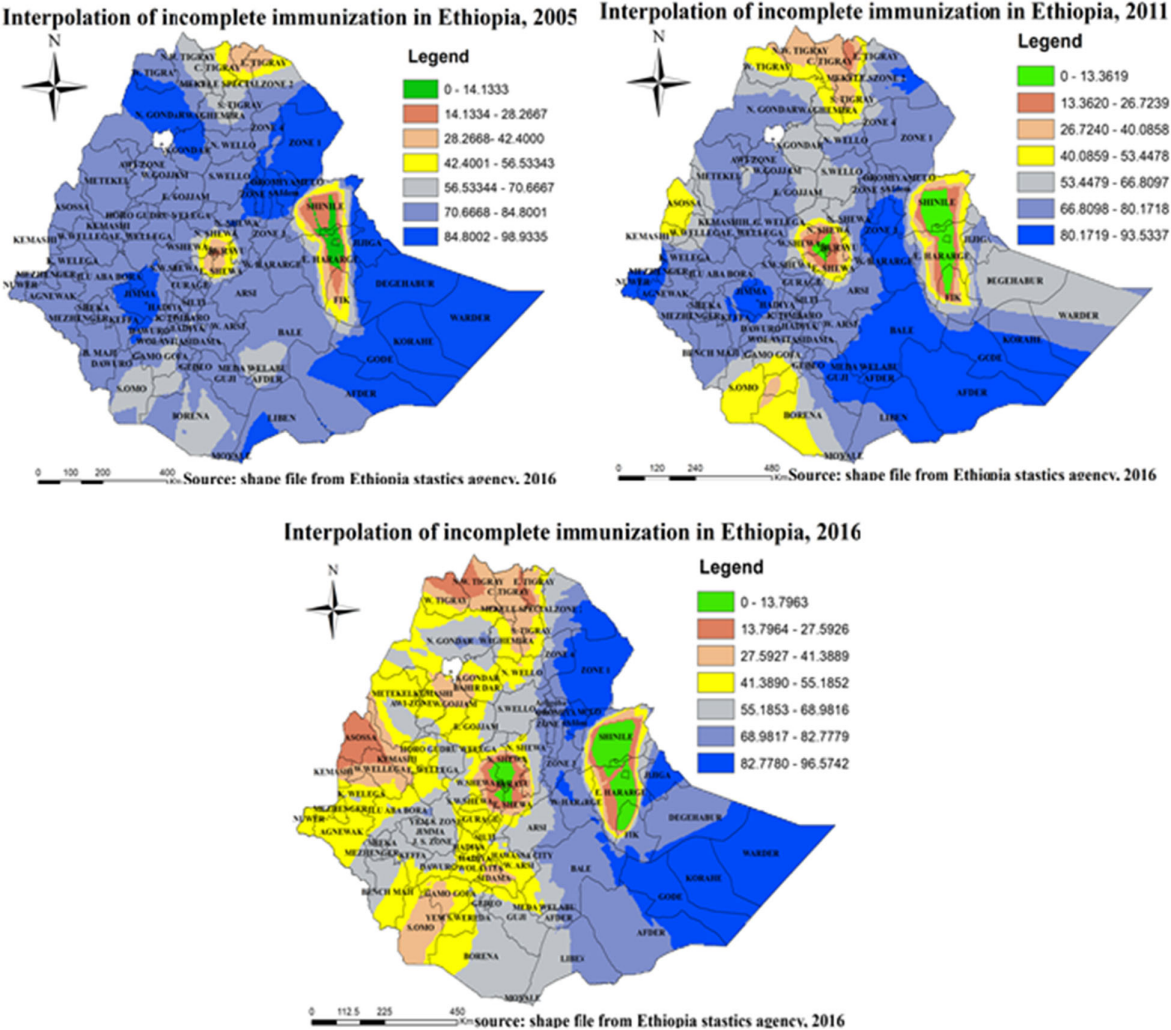
Spatial interpolation of incomplete immunization in Ethiopia, EDHS 2005, 2011 and 2016. Kriging interpolation analysis result of incomplete immunization in Ethiopia from Ethiopian demographic and health survey. The right panel represents for 2005 EDHS, and the left panel represents for 2011 EDHS the right bottom panel represents for 2016 EDHS. In the figure the blue, bright blue, bright black color indicates high risk zones depending on its intensity, the yellow color indicates moderate risk, and the green, gray, bright color indicates low risk zones of incomplete immunization in Ethiopia. The thin black line indicates zonal borders. [Shape file source: (CSA, 2013; URL: https://africaopendata.org/dataset/ethiopia-shapefiles); Map output: Own analysis using ArcGIS 10.7 Software]

The spatial interpolation analysis using kriging technique predicted well high-risk zones for incomplete immunization. Accordingly, Afder, Gode, Korahe, Jijiga, Degahbur, Liben, and Warder zones of Somali region, Zone 1, Zone 2, Zone 4, and Zone 5 of Afar region, North Gondar, South Gondar, North Shewa, and Oromiya special zones of Amhara region, Iluababor and Jimma zones of Oromiya region, Kefa and Dawuro zones of SNNPR region in 2005,, Afder, Gode, Korahe, and Liben zones of Somali region, Zone 2 and Zone 3 of Afar region, Guji, West Harerge, Bale, Medawalbu, and Jimma zones of Oromiya region, Newuer and Agnewak zones of Gambella region in 2011, Afder, Gode, Korahe, Jijiga, Degahbur, and Warder zones of Somali region, Zone 1, Zone 2, Zone 4, and Zone 5 of Afar region, West Harerge and Bale zones of Oromiya region in 2016 were predicted as high risk regions. On the contrary Shine zone of Somali region, Diredawa city administration, Harari region, East Harerge of Oromiya region in 2005, Shine and Fik zone of Somali region, Diredawa city administration, Harari region, East Harerge of Oromiya region, Addis Ababa city administration in 2011, Shine and Fik zone of Somali region, Diredawa city administration, Harari region, East Harerge and East Shewa of Oromiya region, Addis Ababa city administration in 2016 were predicted as low risk regions (Fig. 8).

#### Spatial scan statistics of incomplete immunization

**Fig. 9:**
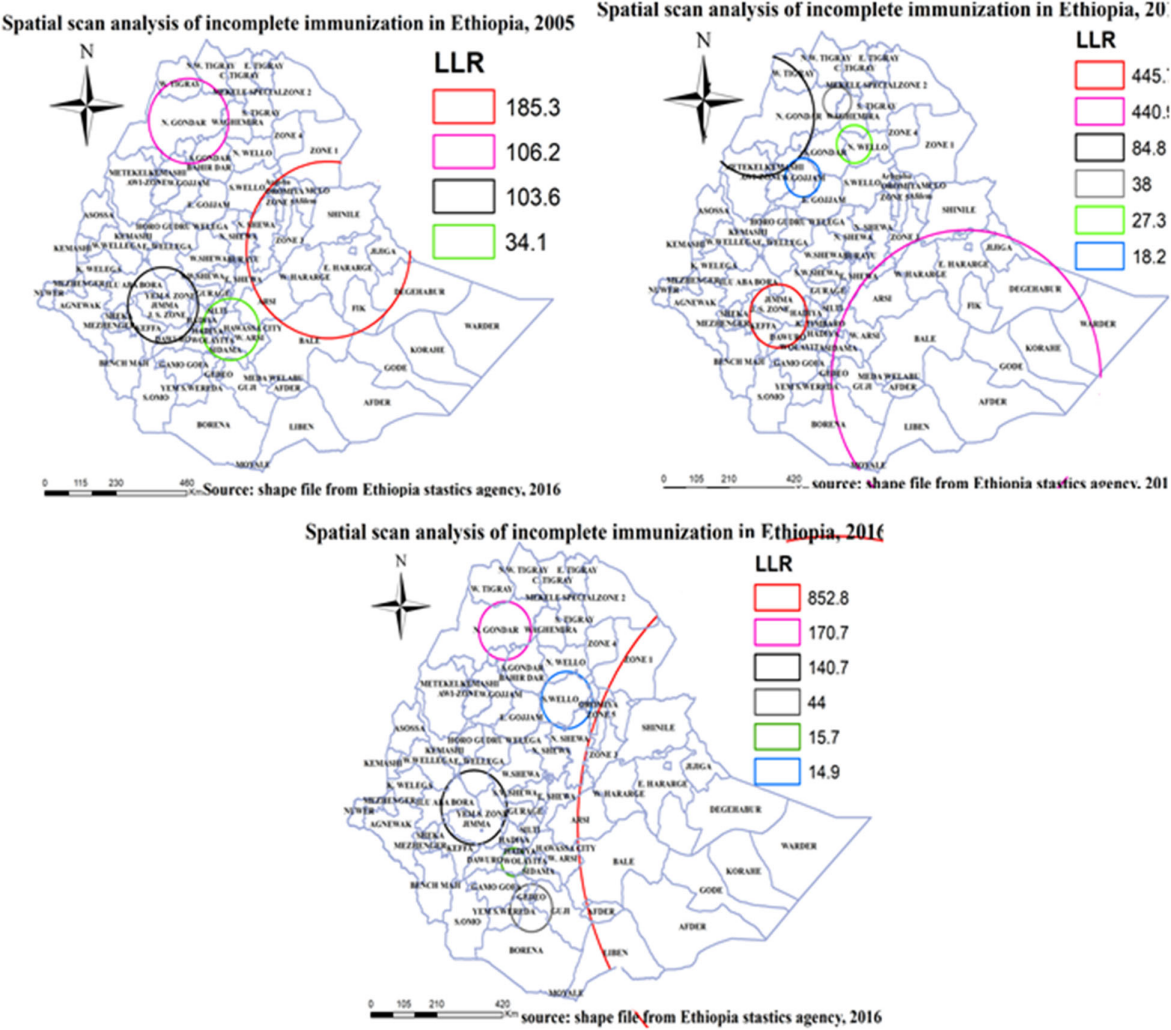
Spatial scan statistics analysis of incomplete immunization in Ethiopia, EDHS 2005, 2011 and 2016. Spatial scan statistics analysis result of incomplete immunization in Ethiopia from Ethiopian demographic and health survey. The right panel represents for 2005 EDHS, and the left panel represents for 2011 EDHS the right bottom panel represents for 2016 EDHS. In the figure the red color rings indicates primary spatial window, the rose, black, bright black, green and blue colored ring indicates secondary spatial windows. The blue line indicates zonal borders. [Shape file source: (CSA, 2013; URL: https://africaopendata.org/dataset/ethiopia-shapefiles); Map output: Own analysis using SatScan 9.6 Software]

Spatial scan statistics identified primary and secondary clusters of incomplete immunization using the maximum spatial circular windows ≤25% of the total population. Since, spatial scan statistics is strong enough in detecting clusters other than the other methods it acts as a better confirmatory way. Accordingly, spatial scan statistics identified one primary and three secondary clusters in 2005, one primary and five secondary clusters in 2011 and 2016. The primary cluster in 2005 (LLR = 185.3, *P* < 0.001) encompasses Shine, Jijiga, Debgahbur, Fik zones of Somali region, Arsi, Bale, West Harerge, East harerge, and East Shewa zones of Oromiya region, North Shewa, Argoba, and Oromiya special zones of Amhara region, Zone 1, Zone 3, Zone 5 of Afar region, Diredawa city administration and Harari region and it was centered at (9.953773 N, 38.599579 E) with 88.39 km radius and relative risk (RR) of 1.15. In 2011 the primary cluster (LLR = 445.7, P < 0.001) encompasses Jimma, and Iluababora zones of Oromiya region, Kefa and Dawuro zones of SNNPR region and it was centered in (7.486152 N, 36.654976 E) with 91.84 km radius and relative risk (RR) of 1.43. In 2016 the primary cluster (LLR = 852.8, P < 0.001) encompasses Shine, Afder, Gode, Korahe, Jijiga, Fik, Liben, Debgahbur, and Warder zones of Somali region, West Arsi, Arsi, Bale, West Harerge, East harerge, and East Shewa zones of Oromiya region, Zone 1, Zone 3, Zone 5 of Afar region, Diredawa city administration and Harari region and it was centered in (7.717177 N, 46.991581 E) with 814.53 km radius and relative risk (RR) of 1.6 (Fig. 9, Table 4).

**Table 4:** summary of spatial scan statistics results, 2005, 2011, and 2016 EDHS

Year	Detected Clusters	Coordinate /radius	Population	Cases	RR	LLR
2005	Primary cluster**	(9.307287 N, 41.457558 E) / 261.17 km	7363	6315	1.13	185.3
	Secondary cluster**	(9.953773 N, 38.599579 E) / 88.39 km	2464	2186	1.15	106.2
	Secondary cluster**	(7.837178 N, 36.645664 E) / 112.42 km	1697	1539	1.18	103.6
	Secondary cluster**	(7.194395 N, 38.616467 E) / 91.30 km	4882	4013	1.17	34.1
2011	Primary cluster**	(7.486152 N, 36.654976 E) / 91.84 km	2045	1917	1.43	445.7
	Secondary cluster**	(5.842888 N, 42.105068 E) / 431.94 km	8346	6696	1.26	440.9
	Secondary cluster**	(12.706300 N, 36.064266 E) / 176.87 km	3026	2345	1.15	84.8
	Secondary cluster**	(13.059921 N, 38.362839 E) / 44.40 km	879	705	1.2	38
	Secondary cluster**	(11.964659 N, 38.856392 E) / 55.04 km	488	400	1.1	27.3
	Secondary cluster**	(11.090950 N, 37.365948 E) / 55.98 km	1200	900	1.12	18.2
2016	Primary cluster**	(7.717177 N, 46.991581 E) / 814.53 km	4924	4025	1.6	852.8
	Secondary cluster**	(12.673719 N, 37.510685 E) / 81.11 km	1801	1378	1.39	170.7
	Secondary cluster**	(8.246355 N, 36.621498 E) / 103.16 km	1358	1053	1.4	140.7
	Secondary cluster**	(5.725346 N, 38.264767 E) / 66.28 km	684	502	1.31	44
	Secondary cluster**	(6.876749 N, 37.757759 E) / 38.81 km	960	624	1.16	15.7
	Secondary cluster**	(10.939576 N, 39.281273 E) / 79.57 km	648	432	1.19	14.9

** = *p* < 0.0001

### Spatial regression analysis

#### Ordinary least square

**Fig. 10:**
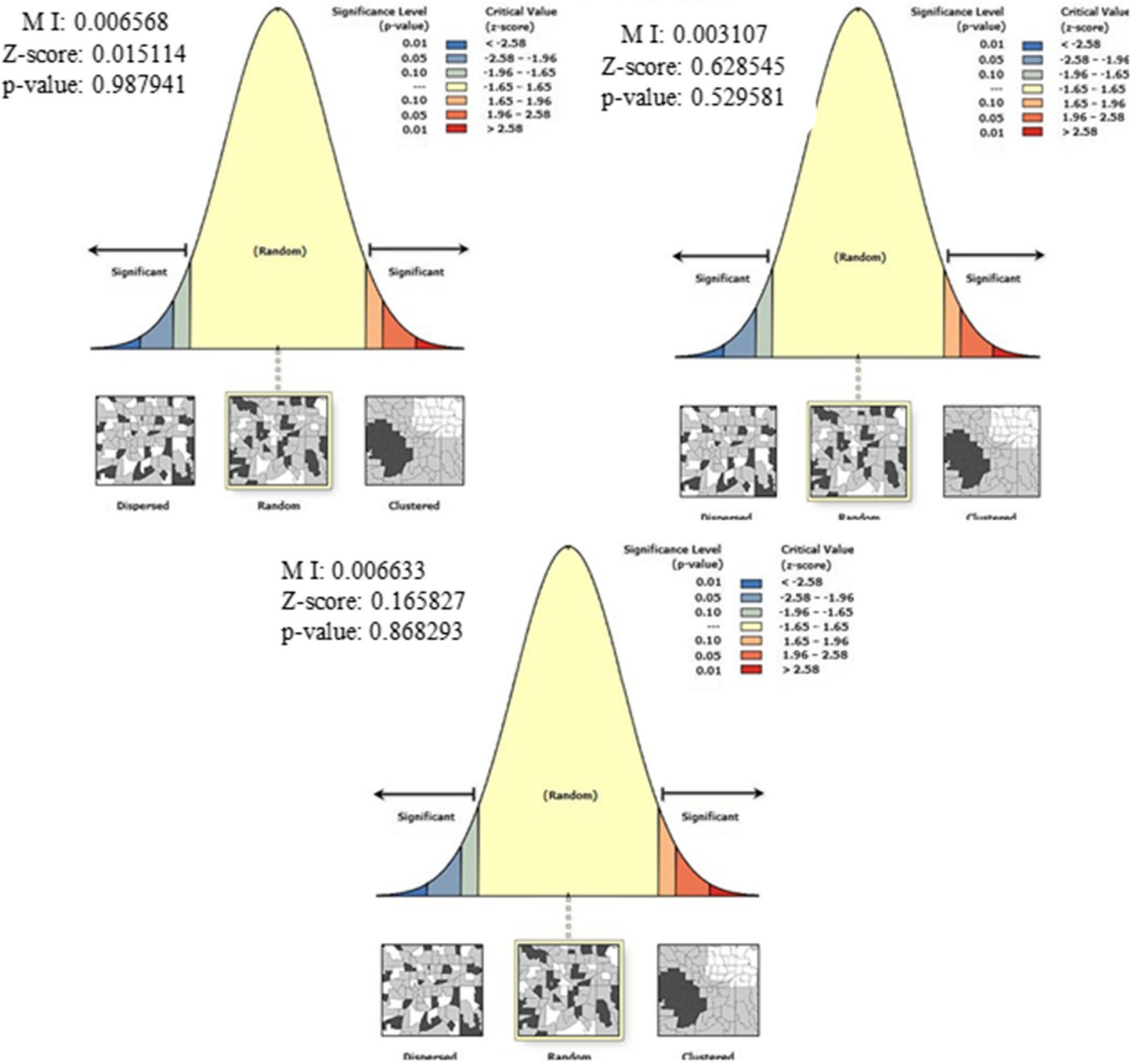
Spatial analysis of Geographic Weighted Regression in Ethiopia, EDHS 2005, 2011 and 2016 EDHS 2005, 2011 and 2016. The right panel represents for 2005 EDHS, the middle panel represents for 2011 EDHS, and the left panel represents for 2016 EDHS. The right part of the panel indicates dispersed, the central part of the panel indicates random, and the left part of the panel indicates clustered. In this study, residuals of GWR are distributed randomly in all periods. The random pattern on the middle side of the panels indicates that spatially uncorrelated residuals in GWR analysis of the study periods. The output has automatically generated keys on the upper right side of the panel as well as in the underneath reveals that; the pattern does not appear to be significantly different than random. [Shape file source: (CSA, 2013; URL: https://africaopendata.org/dataset/ethiopia-shapefiles); Map output: Own analysis using ArcGIS 10.7 Software]

After checking spatial regression assumptions for each survey (2005, 2011, 2016), exploratory regression with ordinary least square analysis was carried out. Variables which have consistency of significance 60% and above were selected for ordinary least square analysis. Outputs from the spatial regression analysis revealed that, residuals of spatial relationship are uncorrelated (Fig. 10, Table 5, Table 6 and Table 7) and there was no multicollinearity among explanatory variables (Table 8, Table 9, Table 10). In order to handle geographical weighted regression (the koenker Bp test) in the ordinary least square analysis could be significant, which reveals the difference of coefficients across enumeration areas. In the context of this finding koenker, Bp test is significant therefore executing geographically weighted regression is possible.

**Table 5:** spatial regression summary result of ordinary least square (global GWR) diagnostics for 2005 EDHS data

Diagnostics criteria	magnitude	p-value
AICc	5428.48	
R squared	0.27312	
Adjusted R squared	0.267543	
Joint f statistics	49.12	0.0000*
Joint wald statistics	288.95	0.0000*
Koenker (Bp) statistics	40.14	0.0000*
Jareque-Bera statistics	11.77	0.2673

**Table 6:** Spatial regression summary result of ordinary least square (global GWR) diagnostics for 2005 EDHS data

Diagnostics criteria	magnitude	p-value
AICc	5504.0537	
R squared	0.267156	
Adjusted R squared	0.261628	
Joint F statistics	48.3	0.0000*
Joint Wald statistics	247.72	0.0000*
Koenker (Bp)statistics	12.622	0.0000*
Jareque Bera statistics	8.34	0.2673

**Table 7:** Spatial regression summary result of ordinary least square (global GWR) coefficients for 2016 EDHS data

Diagnostics criteria	magnitude	p-value
AICc	5994.537	
R squared	0.36087	
Adjusted R squared	0.356721	
Joint f statistics	87.090497	0.0000*
Joint Wald statistics	472.091699	0.0000*
Koenker (Bp)statistics	7.259042	0.02262*
Jareque-bera statistics	3	0.18411

**Table 8:** Spatial regression summary result of ordinary least square (global GWR) coefficients for 2005 EDHS data

Variables	coefficient	Probability	Robust Probability	Vif
Intercept	10.7617	0.0023	0.00009	
No education	0.308165	0.0000	0.0000	1.4
Mothers Aged 15–24	0.180752	0.000034	0.000095	1.3
Poor wealth index	0.220214	0.000742	0.000447	1.2
Male household head	0.159098	0.001784	0.0003068	1.2

**Table 9:** Spatial regression summary result of ordinary least square (global GWR) coefficients for 2011 EDHS data

Variables	Coefficient	Probability	Robust Probability	Vif
Intercept	12.017139	0.000534	0.00002	
No education	0.286607	0.0000	0.0000	1.5
Muslim	0.110434	0.01497	0.029645	1.4
Poor wealth index	0.195264	0.000235	0.00022	1.08
Male household head	0.183557	0.00069	0.00069	1.2

**Table 10:** Spatial regression summary result of ordinary least square (global GWR) coefficients for 2016 EDHS data

Variables	Coefficient	Probability	Robust-Probability	Vif
Intercept	8.07139	0.001432	0.00019	
Not education	0.25638	0.0000	0.0000	1.5
Poor wealth index	0.31749	0.0000	0.0000	1.4
Not working	0.1749	0.0001	0.0005	1.08
Having Children 4–6	0.0435	0.3782	0.4372	1.2

Accordingly, we have executed geographically weighted regression and determined local coefficients of each independent variable.

#### Interpretations of ordinary least square results

In EDHS 2005 ordinary least square analysis: mother’s age (15–24 years), non-educated respondents, poor wealth index and male household head were identified as predictors for incomplete immunization. A unit change in mothers age (15-24 years), poor wealth index and male household head increases the expected log odds of incomplete immunization by 0.180752, 0.220214 and 0.159098 respectively (Table 8).

In EDHS 2011 ordinary least square analysis: not education, wealth index, religion and male household head were significantly associated with incomplete immunization. A unit increase for non-educated respondents, Muslim religion followers, poor wealth index and male household head increases the expected log odds of incomplete immunization by 0.286607, 0.110434, 0.195264 and 0.183557 respectively (Table 9).

Similarly, in EDHS 2016 ordinary least square analysis: non educated, mother’s with poor wealth index and mothers who do not have occupation were significant predictors. A unit increase for respondents who were not-educated, respondents who do not have occupation and poor wealth index increase the log odds on incomplete immunization by 0.5638, 0.1749 and 0.31749 respectively (Table 10).

#### Results for geographically weighted regression

**Fig. 11:**
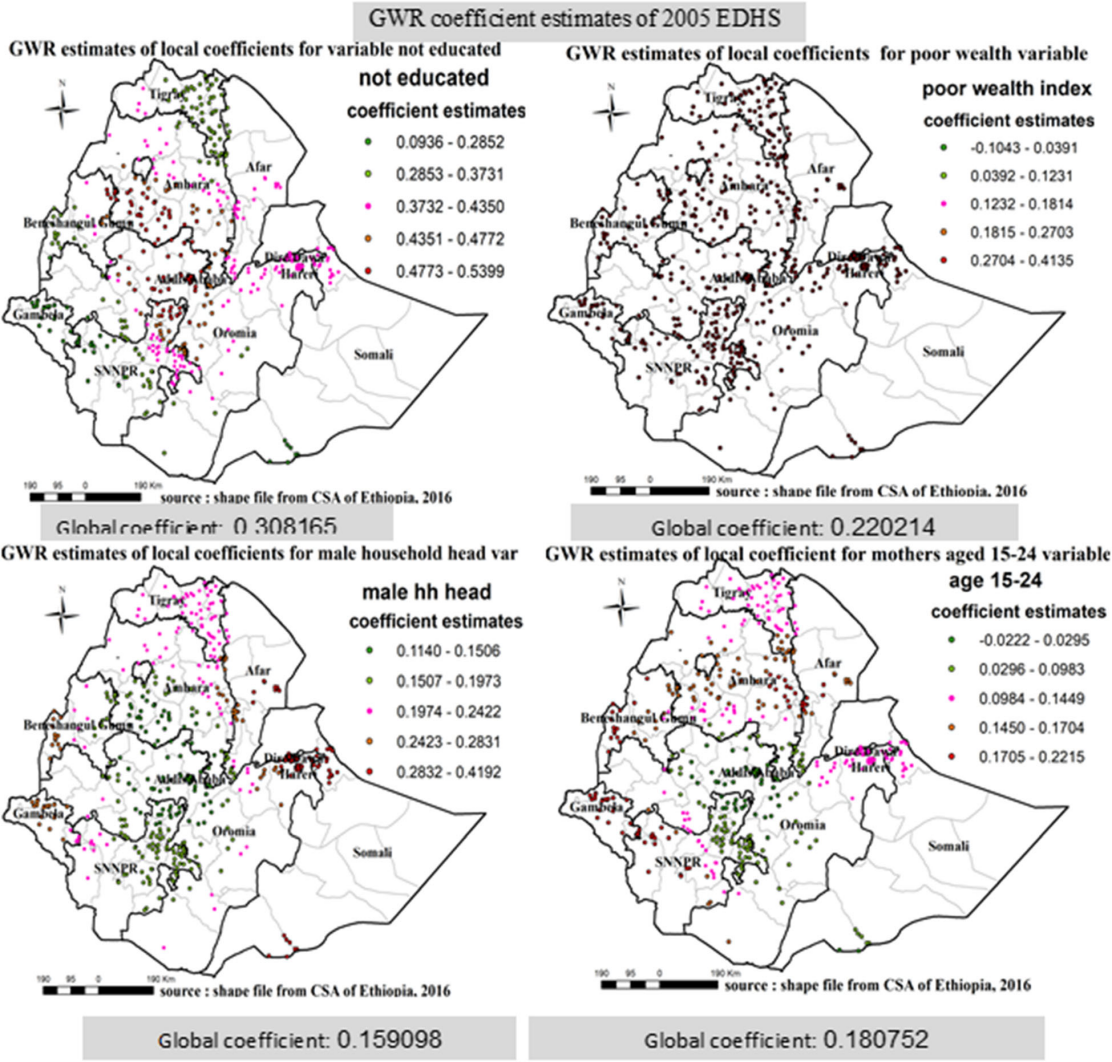
Geographic Weighted Regression analysis in Ethiopia, EDHS 2005 EDHS 2005, 2011 and 2016. Geographically weighted regression (GWR) analysis result of explanatory variables (not educated, poor wealth index, mothers aged 15–24, and male household head) with incomplete immunization in Ethiopia from Ethiopian demographic and health survey 2005. Each point in the map represents one enumeration area which has a lots of incomplete immunization cases. The right panel represents for not educated, the left panel represents for poor wealth index, and the right bottom panel represents for male household head and the right bottom panel represents for mothers aged 15–24. In the figure the bright red color indicates high magnitude of coefficient estimates, the green color indicates low magnitude of coefficient estimates, and the rose color indicates medium magnitude of coefficient estimates. The thick black line indicates regional borders while the thin white black line indicates zonal borders. [Shape file source: (CSA, 2013; URL: https://africaopendata.org/dataset/ethiopia-shapefiles); Map output: Own analysis using GWR 4.0 Software]

**Fig. 12:**
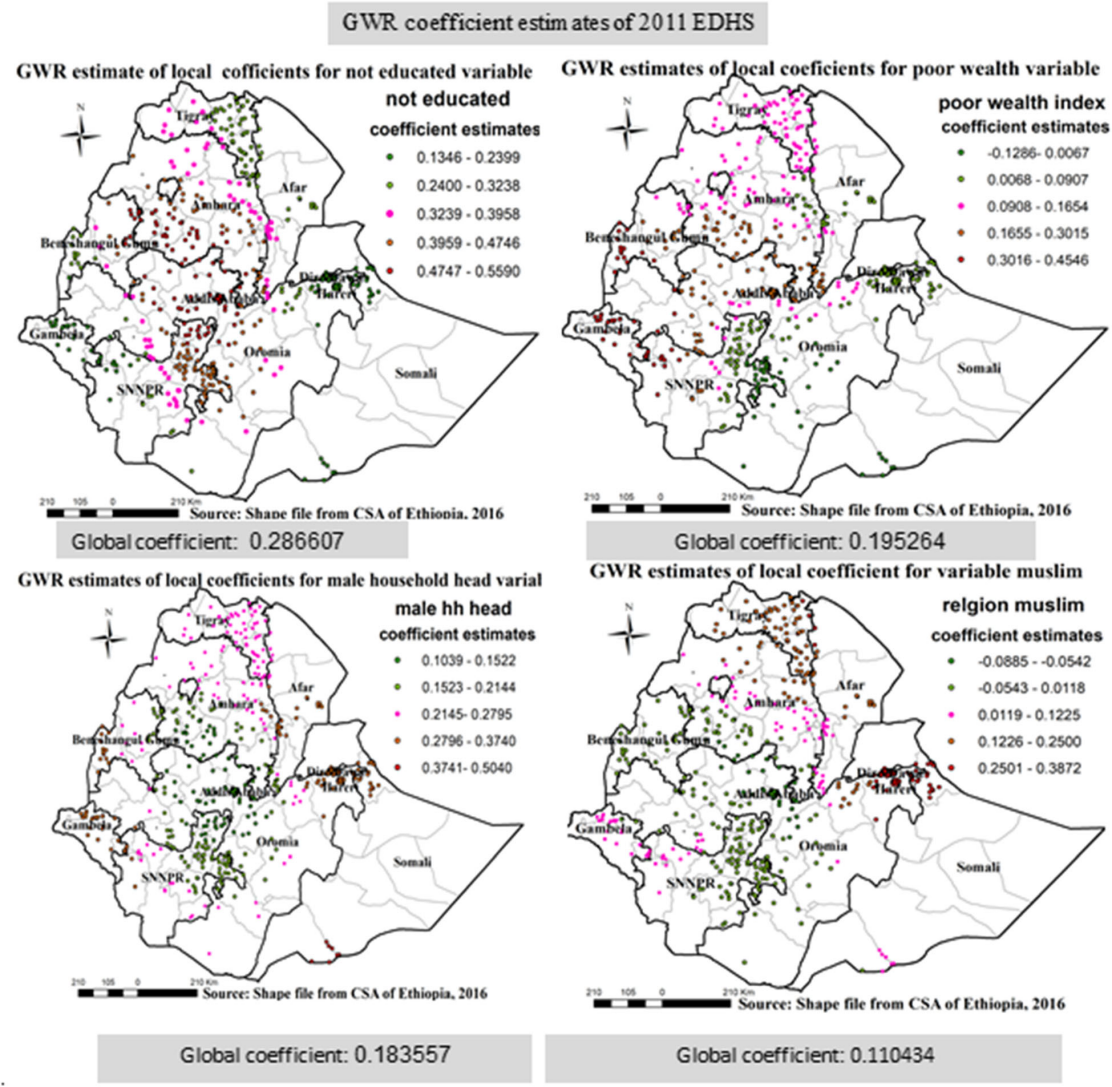
Geographic Weighted Regression analysis in Ethiopia, EDHS 2011 EDHS 2005, 2011 and 2016. Geographically weighted regression (GWR) analysis result of explanatory variables (not educated, poor wealth index, Muslim religion, and male household head) with incomplete immunization in Ethiopia from Ethiopian demographic and health survey 2011. Each point in the map represents one enumeration area which has a lot of incomplete immunization cases. The right panel represents for not educated, the left panel represents for poor wealth index, and the right bottom panel represents for male household head and the right bottom panel represents for Muslim religion. In the figure the bright red color indicates high magnitude of coefficient estimates, the green color indicates low magnitude of coefficient estimates, and the rose color indicates medium magnitude of coefficient estimates. The thick black line indicates regional borders while the thin white black line indicates zonal borders. [Shape file source: (CSA, 2013; URL: https://africaopendata.org/dataset/ethiopia-shapefiles); Map output: Own analysis using GWR 4.0 software]

**Fig. 13:**
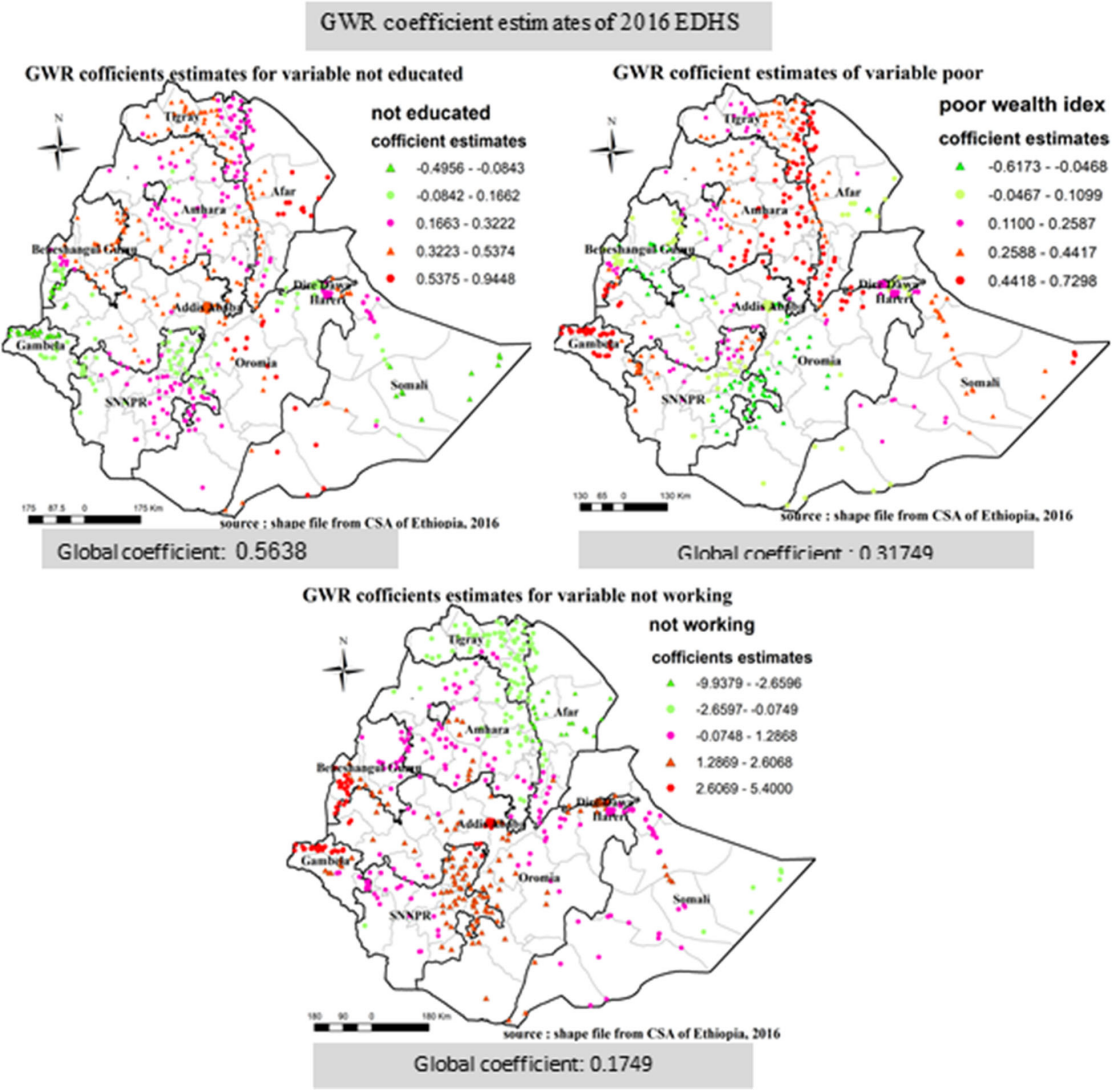
Geographic Weighted Regression analysis in Ethiopia, EDHS 2016 EDHS 2005, 2011 and 2016. Geographically weighted regression (GWR) analysis result of explanatory variables (not educated, poor wealth index, and not working) with incomplete immunization in Ethiopia from Ethiopian demographic and health survey 2016. Each point in the map represents one enumeration area which has a lot of incomplete immunization cases. The right panel represents for not educated, the left panel represents for poor wealth index, and the right bottom panel represents for not working. In the figure the bright red color indicates high magnitude of coefficient estimates, the green color indicates low magnitude of coefficient estimates, the rose color indicates medium magnitude of coefficient estimates, the thick black line indicates regional borders while the thin white black line indicates zonal borders. [Shape file source: (CSA, 2013; URL: https://africaopendata.org/dataset/ethiopia-shapefiles); Map output: Own analysis using GWR 4.0 Software]

The result of geographical weighted regression identified different variable coefficients in different parts of the country for those identified variables on ordinary least square analysis for all the three consecutive surveys; in 2005, Southern parts of Amhara, Central parts of Oromia and Northern parts of SNNPR region, Ganbela and Western parts of Benshangul region, parts of Somali nearby Dire Daw, Dire Dawa and Harari city administration, North Western SNNPR, Eastern Amhara, Ganbela and Western parts of Benshangul region was area which has higher coefficient for mothers who are not educated, mothers who has poor wealth index, male household head and mothers aged 15–24 variables respectively (Fig. 11). In 2011, Southern parts of Amhara, Central parts of Oromia and Northern parts of SNNPR region, Ganbela, Dire Dawa and Harari city administration, Eastern Amhara, parts of Somali nearby Dire Daw, Dire Dawa and Harari city administration was area which has higher coefficient for mothers who are not educated, mothers who has poor wealth index, male household head and mothers who has Muslim religion variables respectively (Fig. 12). Similarly, In 2016, Central parts of Oromia and Central parts of Afar region, Eastern Tigray, Northern Afar, Southern Afar, Ganbela and Southern parts of Benshangul-Gumuz- regions and Ganbela region were areas of respondents having higher coefficient for mothers who are not educated, has poor wealth index, and have no occupation (Fig. 13).

## Discussion

The issues of incomplete immunization remain a significant problem in Ethiopia even with effort exerted towards immunization by the government of Ethiopia as well as WHO and different organization which works towards child health in the last two decades. The reports of EDHS ensure very stagnant reduction of incomplete immunization which was 65% in 2005 and 45.5% in 2016. The descriptive forms of this study also support this concept even though; incomplete immunization was dropped from 74.6%in 2005 to 55.12% in 2016. The burden of incomplete immunization is still very high and not evenly distributed in the country.

Other countries also show geographic variation in vaccination coverage. The findings incomplete immunization in all the study periods was found to be inconsistent. In 2005 it was inconsistent with studies conducted in Pakistan and reports from Federal Ministry of Health through Health Sector Transformation Plan1, findings in 2011 was inconsistent with studies conducted in Nigeria [17], in Ambo Ethiopia [21], and reports from FMOH through HSTP1, findings in 2016 were inconsistence with studies conducted in Togo [18], Democratic Republic of Congo [19], Ghana [20], South West Ethiopia [22], Wollo Ethiopia [56], Armachiho Ethiopia [23] and reports from FMOH through HSTP1. The probable reason for the discrepancy might be due to; those pocket studies in Ethiopia might not use adequate representative sample size, whereas this study used a national representative data.

The size of a sample determines the precision or level of confidence that we have in a sample estimates. An estimate has an associated level of uncertainty, which depends upon the underlying inconsistency of the data as well as the sample size. The more the variability in the population, the greater the uncertainty is the estimate. Similarly, the larger the sample size the more information we have and so uncertainty could be reduced [57, 58]. In addition, there is potential reporting errors of immunization service across the country either intentional or unintentionally by routine health service reports which acts as immediate impute for health sector transformation plan. These findings also inconsistent with studies conducted in Togo, Nigeria, DRC, Ghana, and Pakistan. The probable explanation for this difference could be due to governmental commitment, health system structure and the health policy they are following. In which different studies conducted on the health system of Ethiopia revealed that, the distribution of health care facility, health care professionals, and immunization campaigns scheduled for across the country is not evenly distributed when it evaluated according to the population of regions and the area of land coverage the region encompasses [59–62].

Different studies also revealed the presence of geographical variation for vaccination. A study conducted in Ethiopia on geographical variation of childhood measles vaccination using 2016 EDHS data found significant clustering pattern of childhood measles vaccination (Global Moran’s I = 0.134, *p*-value < 0.0001). In the study statistically significant clusters, areas with low childhood measles vaccination coverage was detected in Zone 1, Zone 2 and Zone 4 of afar region, Afder, Shabelle, Korah, Doolo, Nogob, Jarar, Fatan administrative zones of Somali region, Nuer and Agnuak Administrative zones of Gambella region [33].

A study conducted in butajira, southern Ethiopia on spatial distribution of under immunization among children found significant cluster of under immunization in East of the Butajira Town, and part of the Kebeles of Bati Lejano to the North and South Mekakelegna Jare demeka [34]. A study conducted in Sub-Saharan Africa on Spatial clustering of measles vaccination coverage among child found spatial heterogeneity in measles vaccination coverage across the region (Global Moran’s I of 0.388, *p* < 0.001). Based on the Anslin local Moran’s I, the study identified statistically significant spatial correlation of low vaccination coverage. The Democratic Republic Congo and Madagascar had clustering of low coverage throughout the country. In other countries, clustering of low-coverage was concentrated in specific geographic areas of East Kenya, North Malawi, North Zambia, South Zimbabwe, and South Mozambique [32].

A study conducted in India on exploring spatial variation in different dose of vaccination revealed that the existence of spatial variation in spatial pattern of BCG, Polio, DPT and Measles vaccination coverage in parts of India. the western part of India (Rajasthan and some of the districts of Gujarat and Maharashtra), north-eastern part (Arunachal Pradesh, Nagaland, Manipur, Mizoram, Assam, Tripura and Meghalaya) and some of the districts from the states of central India like Madhya Pradesh, Uttar Pradesh, and Bihar had lower coverage of every vaccination dose, including full immunization [30].

The most convenient explanation for this spatial variation of incomplete immunization could be as a result of geographical variation in the country which ranges from 4550 m above sea level to 110 m below sea level following this there is considerable infrastructure difference like: road, electricity, water, the distribution of health facility, and health care professionals across regions. In addition there is a difference for distribution of vaccine, culture, socio-demographic characteristics of mothers, attitude and knowledge difference of the society towards immunization across different regions Overall this form of difference in Ethiopia could be came up with inequalities of incompletely immunized children across different parts of the country [59, 60, 62, 63].

In spatial regression analysis, different statistically significant predictor variables of incomplete immunization were identified through the three consecutive study periods in different parts of the country (2005, 2011 and 2016). Being non-educated has poor wealth index, male household head and mothers aged 15–24 in 2005. Being non-educated, has poor wealth index, male household head and Muslim followers in religion in 2011 and being non-educated, has poor wealth index, and mothers currently not working in 2016.

Different studies also revealed that there is considerable significant difference of a certain outcome variable predictors across geographical area [64–66]. This might be due to the reason that there is considerable infrastructure difference among regions which in turn inhibits the educational status of the society. In the context of male household heads; it is directly linked with civilization of the society.

Ideally, fathers and mothers would both be involved and attuned to the health of their children. However, when males are heads of the household, they could not give attention about the vaccination status of the children because they might have different issues that they are going to handle. The intensity of poverty and life style of the people differs from region to region. Poor wealth index could bring incompletely immunized children since people who are poor might not give attention about quality of life.

The analysis of hot spot areas using Getis-Ord spatial auto correlation local Moran’s I and spatial scan statistics fits with the analysis of spatial regression in somewhere and vary also somewhere. The spatial analysis of descriptive statistics was handled using zonal data preference. However, in the analysis of spatial regression we have used point data as immediate source for the analysis.

Therefore, we encountered difficulty to compare the relation between the results of the analysis of hot spot zones with modeling of spatial relationships. This study supports the existing knowledge on the influence of infrastructure coverage, and geographic features on incomplete immunization across the country. This study contributes to identify specific hot spot zones throughout the country and factors significantly affect incomplete immunization, which is very important for intervention. Even though there is documented influence of factors like infrastructure coverage and geographic features it is difficult to understand specific hot spot zones for incomplete immunization in which this study could brought a solution.

### Limitation of the study

There was an enumeration area which had zero latitude and longitude values. Accordingly, it might bring biased estimation of the distribution of incomplete immunization especially in 2005 EDHS. In addition, the location data values were shifted 1-2kms for urban and 10kms for rural areas for data confidentiality issues, consequently, this was the challenge to know the exact cases’ location.

## Conclusion

The trend of incomplete immunization was dropped through the study periods. However, incomplete immunization remains very high, more than half of the children started immunization had not completed even in the recent survey.

The spatial distribution of incomplete immunization was not random; it was clustered in all the study EDHS periods. High risk areas were identified in the three consecutive surveys; Kefa, Gamogofa, Kembata Temibaro, and Hadya zones of SNNPR region, Jimma zone of Oromiya region in 2005, Kefa, Gamogofa, Kembata Temibaro, Dawuro, and Hadya zones of SNNPR region, Jimma and West Arsi zones of Oromiya region in 2011, Afder, Gode, Korahe, Warder zones of Somali region In 2016 were detected as significant hot spot regions for incomplete immunization. Therefore, geographically based strategic interventions according to the identified clusters (high-risk areas) could be done for the reduction of incomplete immunization in Ethiopia.

In modeling spatial relationship being not educated, has poor wealth index, and has Muslim religion in 2005, being not educated, has poor wealth index, and has Muslim religion in 2011, being not educated, has poor wealth index, and has Muslim religion in 2016 was significant predictors of incomplete immunization in different parts of the country.

## Data Availability

All relevant data are in the manuscript. However, the minimal data underlying all the findings in the manuscript will be available upon request by contacting the principal investigator via mequsharew8@gmail.com

## Abbreviations

LLR: Log Likelihood Ratio
GIS: Geographic information system
GPS: Global positioning system
SNNPR: Southern nation and nationalities people representatives
EDHS: Demographic and health survey
ICF: International Classification of Functioning, Disability, and Health
RRs: Relative risks
EPI: Expanded Programme of Immunization
EHNRI: Ethiopian Health and Nutrition Research Institute
BCG: Bacilli of Calamite and Gurin
CSA: Central Statistical Agency
EAs: Enumeration Areas
HMIS: Health Management Information System
EFY: Ethiopian Fiscal Year
HSTP1: Health Sector Transformation Plane One
FMOH: Federal Ministry of Health
GWR: Geographic Weighted Regression
OLS: Ordinary Least Square
